# The Relation of Syphilis to the Practice of Dentistry

**Published:** 1891-01

**Authors:** 


### The Relation of Syphilis to the Practice of Dentistry.

The number of recorded cases of communicated syphilis occurring in the practice of dentistry are relatively few compared to the other innocent channels through which it has been acquired; still there are sufficient cases on record to show that it has frequently occurred. In the enormous majority of instances syphilis is acquired by sexual intercourse, because here is offered the greatest opportunity for abrasions to occur, through which the poison may gain entrance. But on the other hand, hundreds, or even thousands of physicians and midwives have contracted syphilis in the practice of their calling. A striking illustration is found in the history of eustachian catheterization, where as many as sixty cases were traced to one person.

For the entrance of the syphilitic virus a broken epithilial or epidermal surface is necessary, although apparent exceptions to this rule have been occasionally met. In some way the virus must come in contact with the absorbing elements of the body, either blood vessels or lymphatics, and by them be taken into the circulation where it increases and manifests its lesions in different parts of the body. When once the virus has gained entrance the individual is syphilitic.

The virus may be received from four different sources. *First.*—From the initial sore or chancre. *Second.*—From mucous patches. *Third.*—From syphilitic ulceration. *Fourth.*—From the blood. The second source of contagion, namely, mucous patches, is the one to be careful of in dental practice. At one time or another these lesions appear to a greater or less extent on the buccal mucous membrane of almost every syphilitic person. Mucous patches are slightly raw surfaces of various sizes and shapes, which are at first slightly elevated, and then may become depressed by the loss of epithelial covering. When newly developed they are redder in color than the normal mucous membrane, but later may become grayish-white.

We will now mention a few cases reported. A gentleman in September, 1884, visited a physician, Dr. L. D. Bulkley, of New York City, to see him about what he thought was a cancer on his tongue, but which proved to be a chancre, the characteristic syphilitic eruption following shortly after. About a month previous to his calling on Dr. Bulkley, he had had some dental operation performed, and he said he remembered that the instrument occasionally slipped, and his tongue was injured. The dentist did not appear very clean or particular about his instruments. As he was a married man no other cause could be found by which he could have become affected. Sir William Watson published a case of this description and John Hunter relates two similar cases, about which there can be no doubt. J. C. Lettsom has recorded three cases.

Dr. C. W. Dulles, of Philadelphia, reports a case which was also seen by the late Dr. Maury. The patient, a female domestic of excellent character, developed a chancre of the lip two weeks after a visit to a dentist, who on that occasion extracted a tooth, and later did some cleansing of the teeth. Although the case was hard to confirm it seemed reasonable to suppose that the operation was in some way responsible for the inoculation.

Dr. F. N. Otis, also mentions a chancre of the lip which occurred in a gentleman about three weeks after a visit spent in a dentist's chair; Lancereaux relates a similar case of chancre of the lower lip of a woman after extraction of a tooth and other dental work; and Giovannie, of Bologna, has reported a chancre on the lip from a dentist's instrument; Leloir mentions one from the same cause; Lydston has likewise reported a case of a woman with syphilis, the chancre having developed three weeks after having some cleaning and repairing done to her teeth; Roddick, of Montreal, reports a very interesting case—the patient was the wife of a physician and the mother of healthy children. She became infected while having a tooth extracted.

There are also a number of reported cases where the dentist was the one to suffer, having become inoculated while operating for syphilitic patients; some receiving the virus through an abrasion on the finger, others by scratching the skin on a sharp tooth, or by being bitten by the patient.—*The Dental Mirror.*

